# Supplementary material for: Knowledge, attitude, and practice toward genetic testing in breast cancer patients in China
Source: PLoS One. 2025 May 8;20(5):e0322526. doi: 10.1371/journal.pone.0322526 (PMC12061185; doi:10.1371/journal.pone.0322526)
Supplement: S1 Table — (DOCX) [file pone.0322526.s001.docx]

**Supplement Table S1.** SEM results

|  |  |  | **β** | **P** |
| --- | --- | --- | --- | --- |
| Attitude | <--- | Knowledge | 0.343 | <0.001 |
| Practice | <--- | Attitude | 0.942 | <0.001 |
| Practice | <--- | Knowledge | 0.036 | 0.528 |
