# Supplementary material for: Knowledge, attitude, and practice toward genetic testing in breast cancer patients in China
Source: PLoS One. 2025 May 8;20(5):e0322526. doi: 10.1371/journal.pone.0322526 (PMC12061185; doi:10.1371/journal.pone.0322526)
Supplement: S2 Table — (DOCX) [file pone.0322526.s002.docx]

**Supplement Table S2.** SEM model fit.

| **Indicator** | **Reference standard** | **Measured result** |
| --- | --- | --- |
| **CMIN/DF** | 1-3 excellent, 3-5 good | 2.093 |
| **RMSEA** | <0.08 good | 0.043 |
| **IFI** | >0.8 good | 0.950 |
| **TLI** | >0.8 good | 0.941 |
| **CFI** | >0.8 good | 0.950 |
